# Viral Capsid Proteins Are Segregated in Structural Fold Space

**DOI:** 10.1371/journal.pcbi.1002905

**Published:** 2013-02-07

**Authors:** Shanshan Cheng, Charles L. Brooks

**Affiliations:** 1 Department of Computational Medicine and Bioinformatics, University of Michigan, Ann Arbor, Michigan, United States of America; 2 Department of Chemistry, University of Michigan, Ann Arbor, Michigan, United States of America; 3 Department of Biophysics, University of Michigan, Ann Arbor, Michigan, United States of America; UNC Charlotte, United States of America

## Abstract

Viral capsid proteins assemble into large, symmetrical architectures that are not found in complexes formed by their cellular counterparts. Given the prevalence of the signature jelly-roll topology in viral capsid proteins, we are interested in whether these functionally unique capsid proteins are also structurally unique in terms of folds. To explore this question, we applied a structure-alignment based clustering of all protein chains in VIPERdb filtered at 40% sequence identity to identify distinct capsid folds, and compared the cluster medoids with a non-redundant subset of protein domains in the SCOP database, not including the viral capsid entries. This comparison, using Template Modeling (TM)-score, identified 2078 structural “relatives” of capsid proteins from the non-capsid set, covering altogether 210 folds following the definition in SCOP. The statistical significance of the 210 folds shared by two sets of the same sizes, estimated from 10,000 permutation tests, is less than 0.0001, which is an upper bound on the p-value. We thus conclude that viral capsid proteins are segregated in structural fold space. Our result provides novel insight on how structural folds of capsid proteins, as opposed to their surface chemistry, might be constrained during evolution by requirement of the assembled cage-like architecture. Also importantly, our work highlights a guiding principle for virus-based nanoplatform design in a wide range of biomedical applications and materials science.

## Author Summary

Viruses are increasingly viewed not as pathogens that parasitize all domains of life, but as useful nanoplatforms for synthetic maneuvers in a wide range of biomedical and materials science applications. One of the most well-known examples of virus-based nanotools developed so far features viral capsules as therapeutic agents, which protect and deliver drug molecules to targeted disease sites in the human body before the drug molecules are released. In order to optimize these nano-designs to best fulfill their purposes, we first have to understand properties of the constitutive building blocks of these viral containers, so as to rationalize and guide the synthetic modification attempts. Based on the observation that viral shells are functionally unique to viruses, we hypothesize that the structure of the building blocks must also be distinct from generic proteins, given that function follows form. Our computational modeling and statistical analysis support this novel hypothesis, and recognize the folded topology of these ‘Lego’ proteins as a differentiating factor to ensure correct geometry, and consequently, proper tiling into the large complex architecture. Our findings highlight an important design principle: efforts on imparting new functionalities to virus templates should restrain from disrupting the fundamental protein fold.

## Introduction

**Figure 1:**
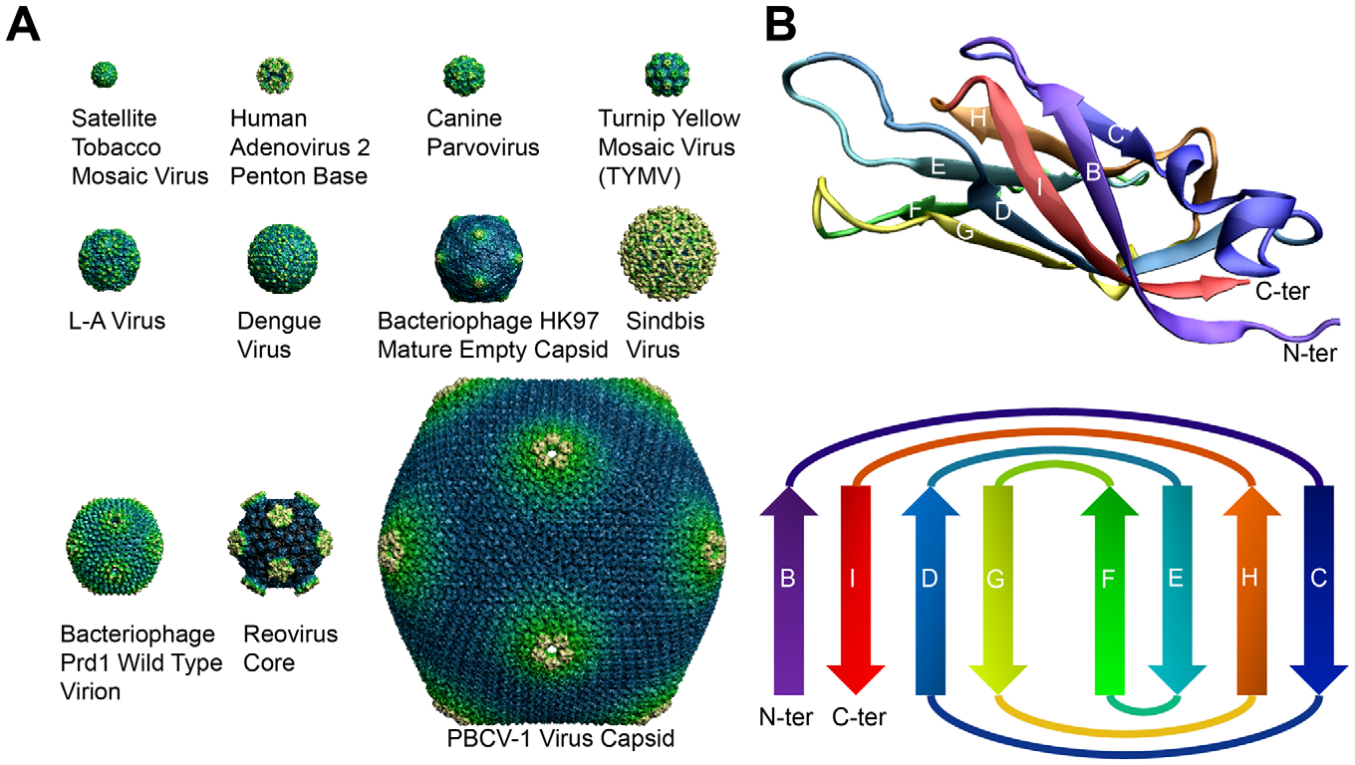
Capsid shells and the folded topology of a typical capsid protein. A) Representative icosahedral viral capsid structures with varying sizes. The Satellite Tobacco Mosaic Virus which is a T = 1 virus has a radius of 8.8 nm, and the Paramecium bursaria Chlorella virus 1 (PBCV-1) which is a pT = 169 virus has a radius of 92.9 nm. Here pT stands for ‘pseudo T number’, which simply means the subunits are not chemically identical (the primary sequences are different). These protein shells are large in that they are assembled from tens of up to hundreds of protein monomers, and they are highly symmetrical. B) The signature jelly-roll of viral capsid proteins, with 8 β-strands forming two antiparallel sheets. The wedge or trapezoidal shape of this particular fold immediately reveals six flat surfaces for monomer-monomer interaction; the sides, the two loop ends and the top and the bottom. The prevalence of the jelly-roll fold among capsid proteins might be related to their relative ease for tiling.

Viral capsid proteins protect the viral genome by forming a closed protein shell around it. Most of currently found viral shells with known structure are spherical in shape and observe icosahedral symmetry [1]. Comprised of a large number of proteins, such large, symmetrical complexes assume a geometrically sophisticated architecture not seen in other biological assemblies. Here we make a distinction between protein cages in viral capsid shells that have sizes ranging from about 10 nm to about 90 nm in radius (Figure 1A), and other oligomeric containers of a much smaller scale, such as ferritins and chaperones. In the simplest form, 60 identical copies of an icosahedral asymmetric unit (IAU) are assembled with 5∶3∶2 symmetry, by positioning three IAUSs on each of the 20 triangular faces of the icosahedron [2]. The triangulation-number, or T-number, can be used to describe the number of proteins in each icosahedral asymmetric unit and therefore the size of the virus. Thus the number of capsid proteins in each shell is a multiple of 60, such as 180 proteins for a T = 3 virus and 240 proteins for a T = 4 virus. While T = 1 viruses can place each protein in an identical environment, other viruses having multiple proteins per IAU achieve the symmetry by following the ‘quasi-equivalence’ principle proposed by Caspar and Klug [3]. Also worth noting is that large viruses, such as double-stranded RNA (dsRNA) viruses, deviate from this principle, while preserving a rigid icosahedral symmetry nonetheless [2].

Geometry of the complex architecture aside, another striking feature of viral capsid proteins lies in the folded topology of the monomers, with the canonical jelly-roll β barrel appearing most prevalent (but not sole) as a core structural motif among capsid proteins that make up these viral shells of varying sizes [4]. Traditionally, this fold has also been termed as a wedge shape [5], an RNA virus capsid domain [6], a β-barrel [7], a β-sandwich [8], and an eight-stranded antiparallel β-barrel fold with a β-roll topology [9], all of which are consistent with the overall morphological characteristic of the fold (Figure 1B). Remarkable diversity in the loop regions connecting the β strands has been observed across different viruses, with variations in length and in inserted segments ranging from secondary structural elements to complete domains [10]. This signature fold of capsid proteins has been extensively studied [11], [12], and has also been compared with non-viral proteins in many separate works, most of which aimed to investigate the evolutionary relationship between viruses and their hosts. Other than the jelly-roll β barrel, there are also the Greek key β barrel with six strands [13], the helix bundle [14] and the immunoglobulin-like fold [15].

Given the unique geometry of the complex formed by viral capsid proteins, one interesting question arises as to whether the structural folds of capsid proteins that assemble into this distinct architecture are also unique to viruses. By comparing the structural topology of capsid proteins that form the icosahedral shells and generic proteins that interact to form other types of complexes, we can potentially establish a link between capsid fold and capsid architecture, or the lack thereof. The answer to this question can lend novel insights to protein-protein interactions, in terms of how folds of protein monomers, as opposed to their surface chemistry, might be related to the assembled multimer complex architecture. Furthermore, the ability of many viral capsid proteins to self-assemble spontaneously makes them an attractive platform for synthetic manipulation across the fields of biomedical applications and nanosciences [16]. Understanding how much influence viral capsid folds place on the assembled architecture is likely to provide guiding principles in the design of drug delivery systems and nanomaterials.

In this work, we present, to the best of our knowledge, the first attempt to examine whether the structural folds of viral capsid proteins set them apart from generic proteins, and with how much statistical significance. We recognize that a general assumption is that any class of proteins with a unique function is expected to be found in exclusive folds, which may or may not hold, given that folded topology is a coarse description of structural characteristics. Thus in addition to testing our hypothesis in the specific case of viral capsid proteins, we perform similar analysis for a few representative classes of proteins with diverse functions. At a finer level of granularity, i.e., the superfamily level, Abroi and Gough also surveyed the classification of all viral proteins and the other superkingdoms to study their genetic interactions in evolutionary history [17]. We distinguish our work by restricting our analysis to viral capsid proteins, which are functionally unique in viruses, in order to establish the link between topology of the building block and the assembled complex architecture. In another related work, Janin and coworkers provided an extensive analysis of physicochemical characteristics of protein-protein interfaces in icosahedral viruses, and compared them with generic protein-protein interfaces [18]. Rather than adopting the same approach of enumerating what's similar and what's different between the two classes, we will employ a direct comparison metric to evaluate whether there is significant statistical evidence supporting our conjecture that viral capsid proteins are structurally unique.

## Materials and Methods

**Figure 2:**
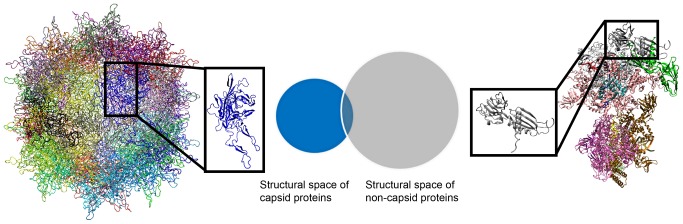
Comparison in structural fold space of capsid proteins and non-capsid ones. Capsid proteins form large, highly symmetric protein shells (left), while generic proteins form other types of complexes (right), exemplified here by an RNA polymerase elongation complex. Overlap between the structural space of viral capsid proteins and that of generic proteins signifies the set of non-capsid ‘relatives’ of capsid proteins. Figure is for illustration purposes and not drawn to scale.

To test our hypothesis that viral capsid folds are not commonly found in generic proteins, we proceed to evaluate if the proportion of non-viral capsid proteins that share similar structural folds with viral capsid proteins is significantly small (Figure 2), based on a well-defined quantitative measure.

### Comparison metric

We chose the Template Modeling-score (TM-score) [19] as our structural comparison metric, for the following reasons. This structure-alignment-based scoring function using the fr-TM-align algorithm [20] is very fast to compute and suits our large-scale comparison; it is normalized, or protein size independent, making the comparison between pairs of domains with complex topology and pairs with simpler ones fair; it has been established in large scale benchmark studies that most of the pairs of proteins with a TM-score of more than 0.5 have the same fold classification, and most of those with a TM-score of less than 0.5 are in different fold classes [21]. In addition, a TM-score of 0.4 has also been extensively used as a criterion to decide if a pair of structures are similar or not [22]. Given that many proteins within the same SCOP fold can have a TM-score of 0.4 and higher, we chose the TM-score of 0.4 as the threshold to validate our hypothesis.

Briefly, the structural alignment score is defined as
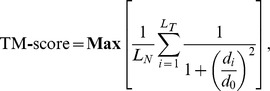
where L_N_ and L_T_ are the lengths of the two peptides being compared, d_i_ is the distance between the C_α_ atoms of the structurally equivalent residues, and d_0_ is a normalization score to make the alignment length-independent. The term *Max* stands for an optimal superimposition between the two structures to minimize distances between structurally-equivalent residues. We define structural distance between a pair of proteins by (1–TM-score), which ranges from zero to one.

### Data collection

In our work, we included all of capsid, nucleocapsid and envelope proteins for analysis, which we collectively call capsid proteins, because of their common structural role in forming the viral shell despite differentiated functions in a few cases. We collected the viral capsid protein set from the VIrus Particle ExploreR (VIPERdb) [23], which is a database of icosahedral virus capsid structures, with 319 entries in total. Altogether 1174 protein chains having at least 80 residues were extracted from these entries, as short peptides are known to assume very simple topologies. These 1174 were further cut into domains; while 452 proteins have domain annotations in SCOP, 637 proteins have homologues (sharing a sequence identity of at least 40%) that are well-annotated by SCOP. The remaining 85 were examined visually and dissected into individual domains. Lastly, the non-compact domains (extended structure with little secondary structure content) are removed, leaving 1447 domains in total.

We used the non-redundant set of 10569 proteins covering 1195 folds from the database Structural Classification Of Proteins (SCOP) 1.75 [24] filtered at 40% sequence identity, available from the ASTRAL compendium [25], to constitute our total protein set. This set was further reduced to 8921 proteins covering 1047 folds after removal of short peptides with fewer than 80 residues. The viral capsid protein set was then subtracted from the total protein set to yield the non-capsid protein set. In addition, 24 capsid proteins in the total protein set that were originally not deposited in VIPERdb were added to the capsid set and removed from the non-capsid set (Table S1). A sequence filter of 40% identity was then applied to the domains of the capsid set, which resulted in 151 domains that are sequence-wise non-redundant.

As viruses across the same family are known to share limited sequence identity despite remarkable structural resemblance, a further structural filter was applied to the capsid set of 151 domains by clustering analysis. We performed hierarchical clustering via the average linkage method, and selected the cluster medoids of the resulting N clusters as our structurally non-redundant capsid set. Optimal partitioning of the data from hierarchical clustering was obtained by choosing the minimal number of clusters such that all intra-cluster distances are less than 0.6, using our structural distance measure. This criterion is based on the rationale that we would like to sort out the most representative capsid structures, without their repeating one another resulting in unfair comparison with the permutation test that we will describe shortly.

**Figure 3:**
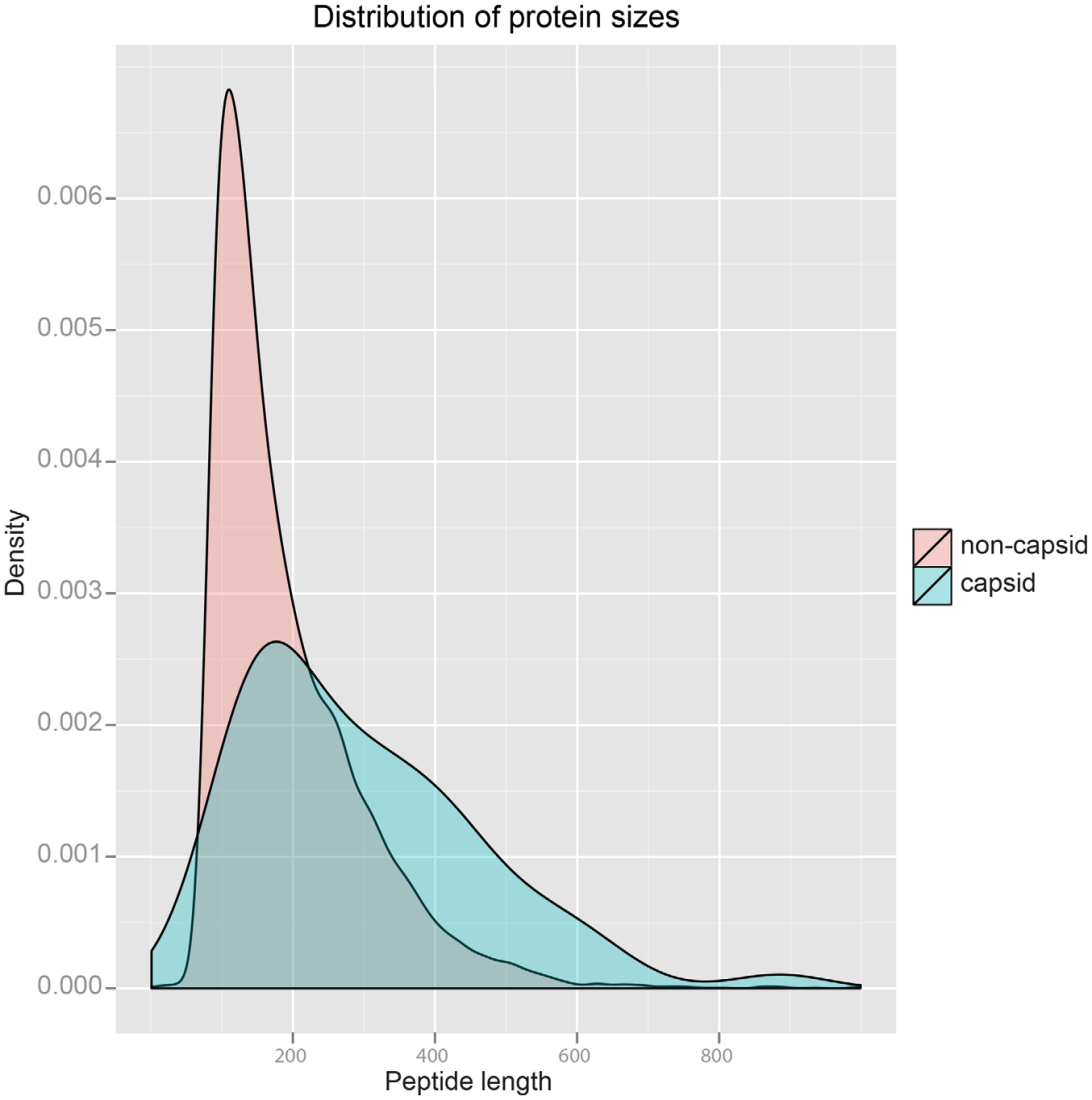
Domain size distribution. Shown in pink is the density distribution of the lengths of non-capsid proteins, and that of capsid proteins is shown in blue. Viral capsid proteins appear to have overall larger domains compared to their cellular counterparts, with a few exceptionally complex domains having more than 600 residues. 600 was later used as a size cutoff in order to examine the two sets that are of comparable sizes.

A preliminary survey of the two sets revealed differences in the sizes of domains. As shown in Figure 3, a typical capsid domain (blue) has approximately 180 residues, compared to about 150 residues for a typical non-capsid domain (pink). This size comparison is purely based on existing structural data of viral capsid proteins, but we do see a larger proportion of complex topologies in certain capsid domains, as opposed to the under-representation of longer folds in generic proteins. In order to preclude the possibility of concluding that capsid and non-capsid proteins have different folds that are in fact largely a result of the difference in length, we performed an additional separate analysis by removing domains having longer than 600 residues in both datasets.

### Shared folds as the test statistic

After obtaining the non-redundant viral capsid set and the non-capsid set, we quantify the extent to which the structural space of the non-capsid set overlaps with that of the capsid set in the following manner. We performed an all-against-all structural comparison between the non-capsid set and the capsid set. For each member in the non-capsid set, we select its nearest neighbor in the capsid set, and use the distance between the two to represent how far structurally this particular non-capsid protein is to viral capsid proteins. With the structural distances between all non-capsid proteins and their nearest neighbors in capsids in hand, we then filter the non-capsid set by retaining only proteins that are less than 0.6 away from capsid proteins. We thus obtain the final distribution of distances between viral capsid proteins and those non-capsid proteins that structurally resemble capsid proteins. Among these ‘relatives’ of viral capsid proteins, we count the number of folds covered by them, following the fold classification in SCOP. This defines our test statistic, which we term as ‘shared folds’ in the rest of the paper.

### Statistical significance of the test statistic

To estimate the statistical significance of the number of shared folds between capsids and non-capsid proteins, we calculated the probability of observing at most the same number of shared folds by random chances by running a permutation test on the total protein set. The total set of proteins was randomly partitioned into set A and set B, with set A consisting of an equal number of proteins as that in the capsid set, and set B being their complement in the total set. The same procedure as described above was carried out to obtain the number of shared folds between this particular set A and their non-self counterparts. To avoid finding ‘relatives’ in set B that are evolutionarily closely related to (i.e. belonging to the same family) the proteins in set A, we further excluded ‘self folds’ from the shared folds found, as an approximation to, or a lower bound of, folds shared with non-self proteins. Here ‘self fold’ is defined as the fold annotation by SCOP of a particular structural analogue found in the large protein set that is already covered by any protein in the small set of proteins. Altogether 10,000 independent permutations were done to give rise to the estimated distribution of shared folds, based on which the p-value of our test statistic can be evaluated.

### Cross-checking with other functional classes of proteins

To examine if unique function generally implies unique folds, we chose a few functional classes of proteins to perform the same analysis described above for capsid proteins. Seven classes were chosen, namely kinases, globins, dehydrogenases, DNA/RNA polymerases, chaperones, antigens and muscle proteins, with functions ranging from catalysis, to transport to signal transduction. The total protein set which is filtered at 40% sequence identity level was partitioned into two sets based on SCOP annotations at the domain level; one being the functional class and the other being the complementary set, and the statistical significance of shared folds is again estimated by permutation tests.

## Results

### Representative folds adopted by viral capsid proteins

**Figure 4:**
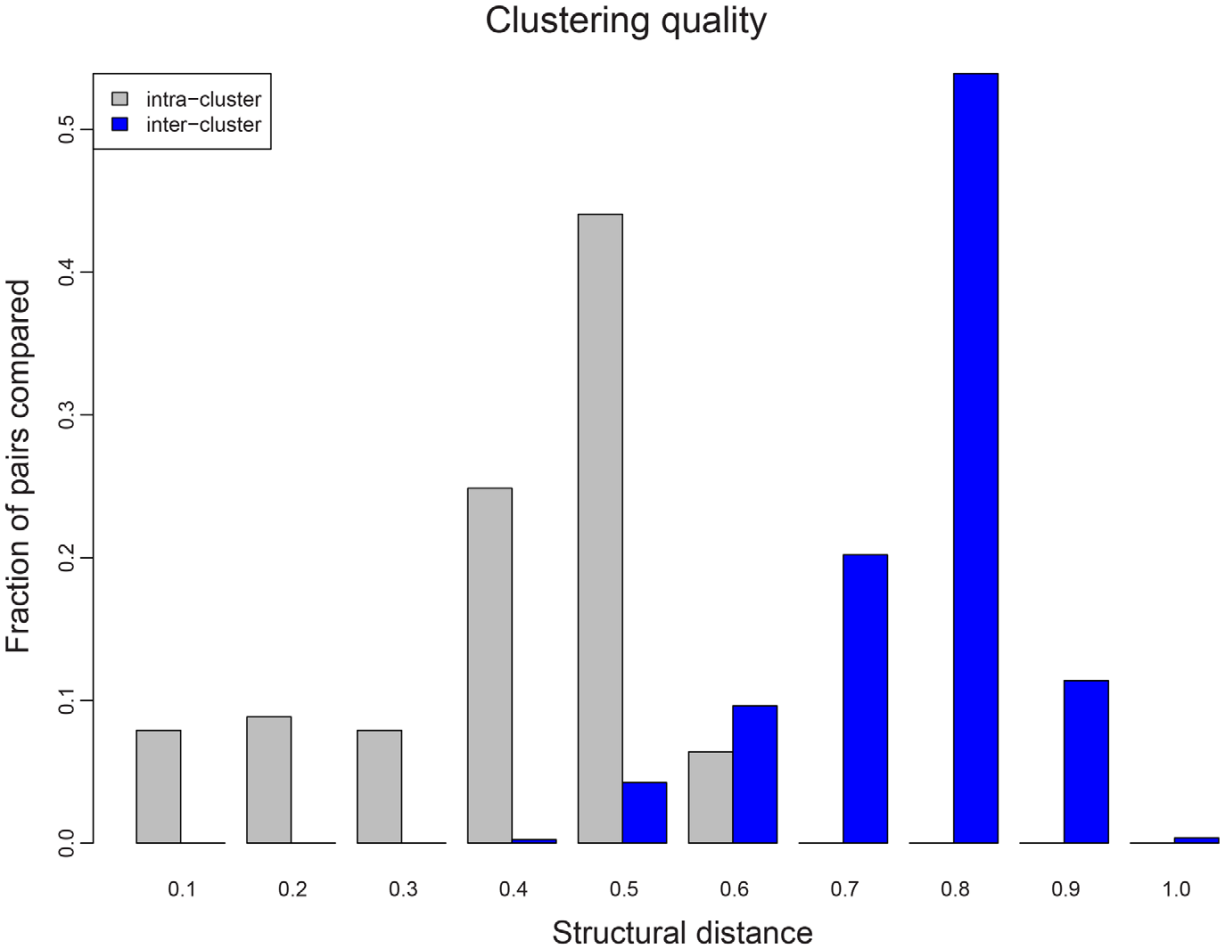
Clustering to find representative capsid folds. Shown here are all pairwise distances between members from the same cluster (grey) and between members from different clusters (blue). Partitioning was chosen such that each cluster is maximally homogeneous, with no members within the same cluster being farther than 0.6 apart.

We found 56 clusters for the viral capsid set, using the criterion described in the Materials and Methods section. These clusters are fairly compact, with all members within each cluster being less than 0.6 apart from one another. Furthermore, the clusters are maximally separated, with only 26 pairs of proteins (0.24%) from two different clusters being closer than 0.4. In Figure 4, we show the statistics demonstrating a good separation between clusters that are reasonably homogeneous. The resulting 56 cluster medoids thus represent the distinct domain architecture adopted by capsid proteins.

**Figure 5:**
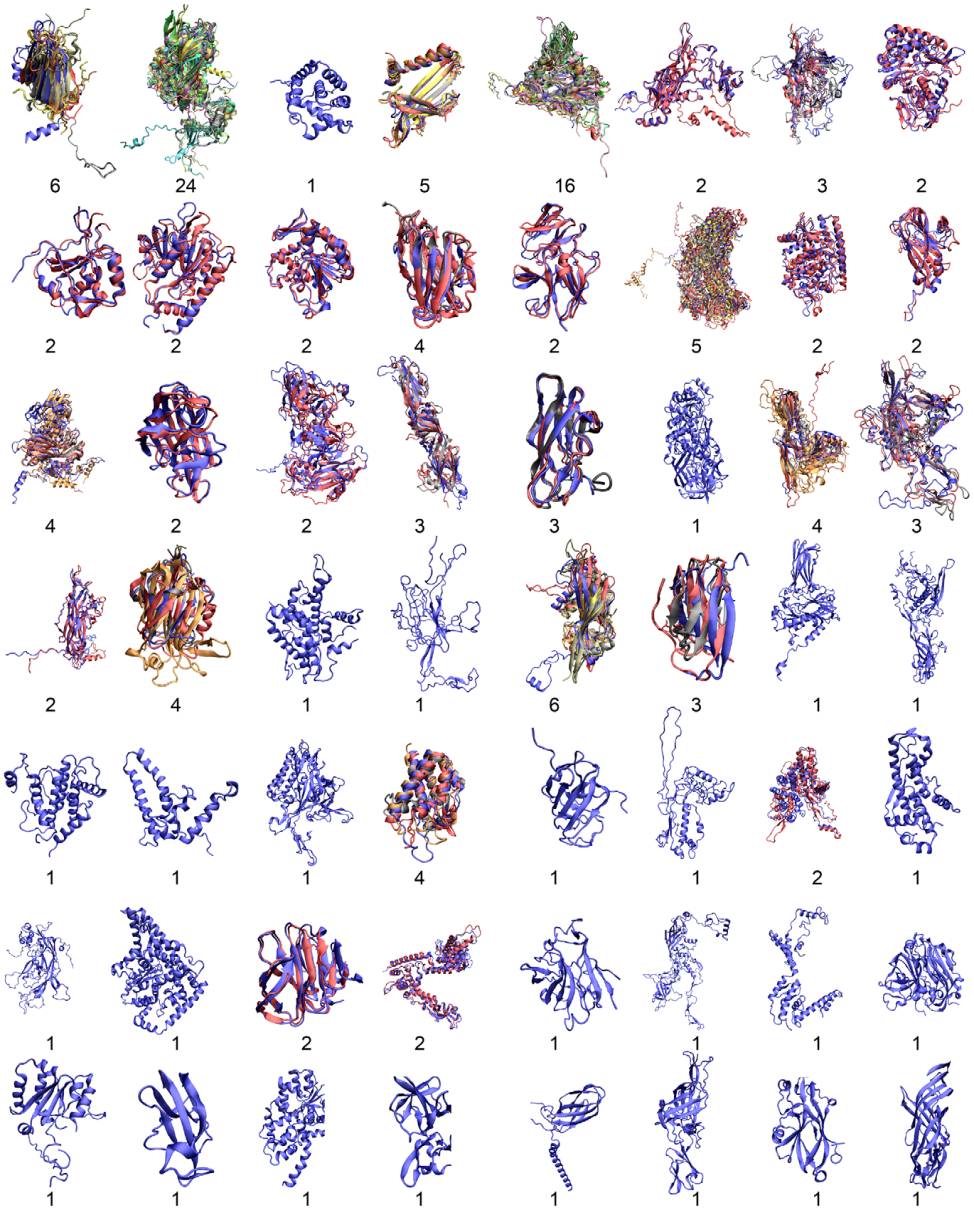
The 56 representative capsid folds. Domains within one cluster are superimposed on one another to show good structural alignment, with number of members in each cluster indicated. The prevalence of singlet clusters reflects the scarcity of structural data for many viral families.

Figure 5 illustrates these 56 clusters with all members in each cluster superimposed on one another. The alignment shows high structural similarity across the same cluster, while different clusters display mostly different folding topologies, in agreement with our quantitative assessment. There are a fairly large number of singlet clusters that are unlike one another, mostly because the structural data for these few viral families are lacking. The few most populated clusters correspond to the canonical jelly-roll fold, with variations in the terminal ends.

### Viral capsid proteins are segregated in structural fold space from generic proteins

**Figure 6:**
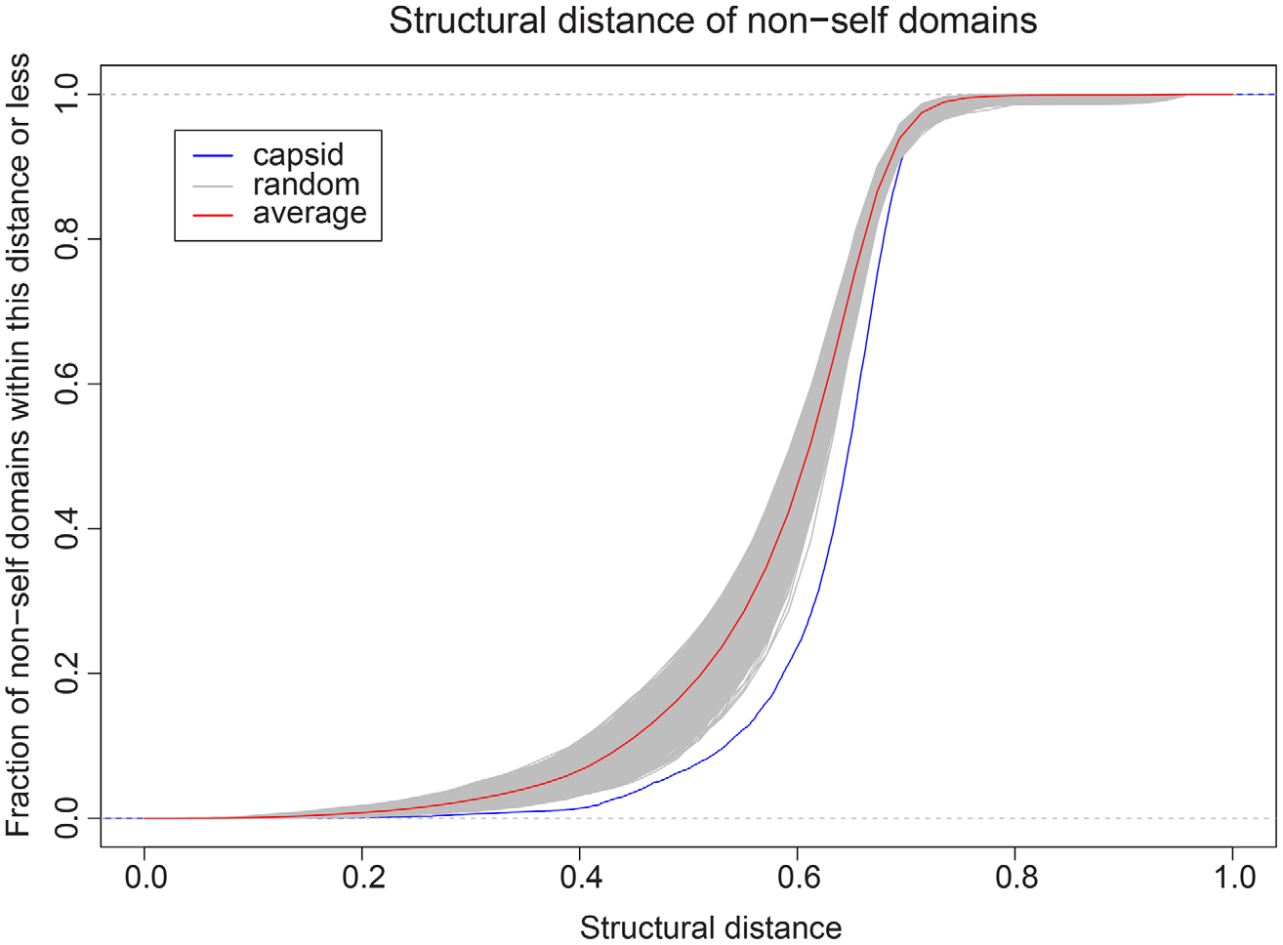
Capsid proteins are structurally distant from generic proteins. Each curve plots the empirical cumulative fraction distribution of distances between one set of 56 proteins and their nearest neighbor in the complementary set. The comparison between the capsid set and the non-capsid proteins is colored in blue, while those from the 10,000 permutation tests are colored in grey. The average empirical cumulative fraction distribution of the 10,000 permutation tests is colored in red. The capsid set is clearly further away from its non-self set compared to what happens with random chances.

By comparing the viral capsid set and the non-capsid set, we found altogether 2078 generic proteins sharing similar topology with viral capsid proteins, based on a distance cutoff of 0.6. These 2078 proteins cover 210 folds in total. If we disregard marginally similar capsid-like proteins by looking at those within a distance 0.5 of capsid proteins only, we find altogether 600 proteins covering 21 folds (Table 1). A further inspection of the distribution of shared folds for randomly sampled sets of 56 proteins and their non-self counterparts immediately reveals that viral capsid proteins are structurally separated from generic proteins. Referring to Figure 6, the cumulative fraction of non-self proteins across the entire structural distance spectrum from viral capsid proteins is clearly shifted to the right compared to those of the 10,000 permutation tests. Through this plot, we expect to arrive at the answer that capsid proteins are different from generic proteins regardless of the distance cutoff used in defining similar folds.

**Table 1:** The 21 folds covered by structural relatives of capsid proteins.

fold (as in SCOP)	name of fold	description of fold	whether contains capsid proteins	example of non-capsid relatives	ID of example
b.1	Immunoglobulin-like beta-sandwich	*sandwich; 7 strands in 2 sheets; greek-key. some members of the fold have additional strands*	Yes	Titin, I27	d1tiua_
b.2	Common fold of diphtheria toxin/transcription factors/cytochrome f	*sandwich; 9 strands in 2 sheet; greek-key; subclass of immunoglobin-like fold*	No	Runt-related transcription factor 1	d1eaqa_
b.6	Cupredoxin-like	*sandwich; 7 strands in 2 sheets, greek-key variations: some members have additional 1–2 strands*	No	Auracyanin	d1qhqa_
b.7	C2 domain-like	*sandwich; 8 strands in 2 sheets; greek-key*	No	Chaperone protein Caf1M	d1p5va2
b.14	Calpain large subunit, middle domain (domain III)	*sandwich; 8 strands in 2 sheets; jelly-roll*	No	M-Calpain	d1df0a2
b.18	Galactose-binding domain-like	*sandwich; 9 strands in 2 sheets; jelly-roll*	No	Xyn10B carbohydrate-binding module	d1h6ya_
b.22	TNF-like	*sandwich, 10 strands in 2 sheets; jelly-roll*	No	Tumor necrosis factor superfamily member 4	d2hewf1
b.23	CUB-like	*sandwich, 10 strands in 2 sheets; jelly-roll*	No	Acidic seminal fluid protein (spermadhesin)	d1sfpa_
b.29	Concanavalin A-like lectins/glucanases	*sandwich; 12–14 strands in 2 sheets; complex topology*	Yes	Sugar binding protein	d1is3a_
b.47	Trypsin-like serine proteases	*barrel, closed; n = 6, S = 8; greek-key duplication: consists of two domains of the same fold*	Yes	human alpha-thrombin	d1h8d.1
b.71	Glycosyl hydrolase domain	*folded sheet; greek-key*	No	alpha-galactosidase	d1uasa1
b.82	Double-stranded beta-helix	*one turn of helix is made by two pairs of antiparallel strands linked with short turns has appearance of a sandwich of distinct architecture and jelly-roll topology*	No	transcriptional regulator, HTH_3 family	d1y9qa2
b.121	Nucleoplasmin-like/VP (viral coat and capsid proteins)	*sandwich; 8 strands in 2 sheets; jelly-roll; some members can have additional 1–2 strands characteristic interaction between the domains of this fold allows the formation of five-fold and pseudo six-fold assemblies*	Yes	Nucleoplasmin-like protein (histone chaperone)	d1nlqa_
b.132	Supernatant protein factor (SPF), C-terminal domain	*sandwich; 8 strands in 2 sheets; jelly-roll; similarity to the Nucleoplasmin-like/VP fold*	No	Lipid Binding Protein	d1olma2
b.135	Superantigen (mitogen) Ypm	*sandwich; 9 strands in 2 sheets; jelly-roll*	No	superantigen from Yersinia pseudotuberculosis	d1pm4a_
c.2	NAD(P)-binding Rossmann-fold domains	*core: 3 layers, a/b/a; parallel beta-sheet of 6 strands, order 321456*	No	Shikimate dehydrogenase	d1nyta1
c.16	Lumazine synthase	*3 layers, a/b/a; core: parallel beta-sheet of 4 strands, order 2134*	No	lumazine synthase	d1ejba_
c.23	Flavodoxin-like	*3 layers, a/b/a; parallel beta-sheet of 5 strand, order 21345*	No	Lysine aminomutase	d1xrsb1
c.37	P-loop containing nucleoside triphosphate hydrolases	*3 layers: a/b/a, parallel or mixed beta-sheets of variable sizes*	No	elongation factor SelB	d1wb1a4
c.44	Phosphotyrosine protein phosphatases I-like	*3 layers: a/b/a; parallel beta-sheet of 4 strands, order 2134*	No	IIBcellobiose	d1iiba_
c.66	S-adenosyl-L-methionine-dependent methyltransferases	*core: 3 layers, a/b/a; mixed beta-sheet of 7 strands, order 3214576; strand 7 is antiparallel to the rest*	No	salicylic acid carboxyl methyltransferase	d1m6ex_

14 out of these 21 folds are either greek-key or jelly-roll (the latter fold being a specific variation of the former). Remarkably, 17 folds are specific to non-capsid proteins, and are only marginally similar to capsid proteins in structure.

### Estimation of statistical significance

**Figure 7:**
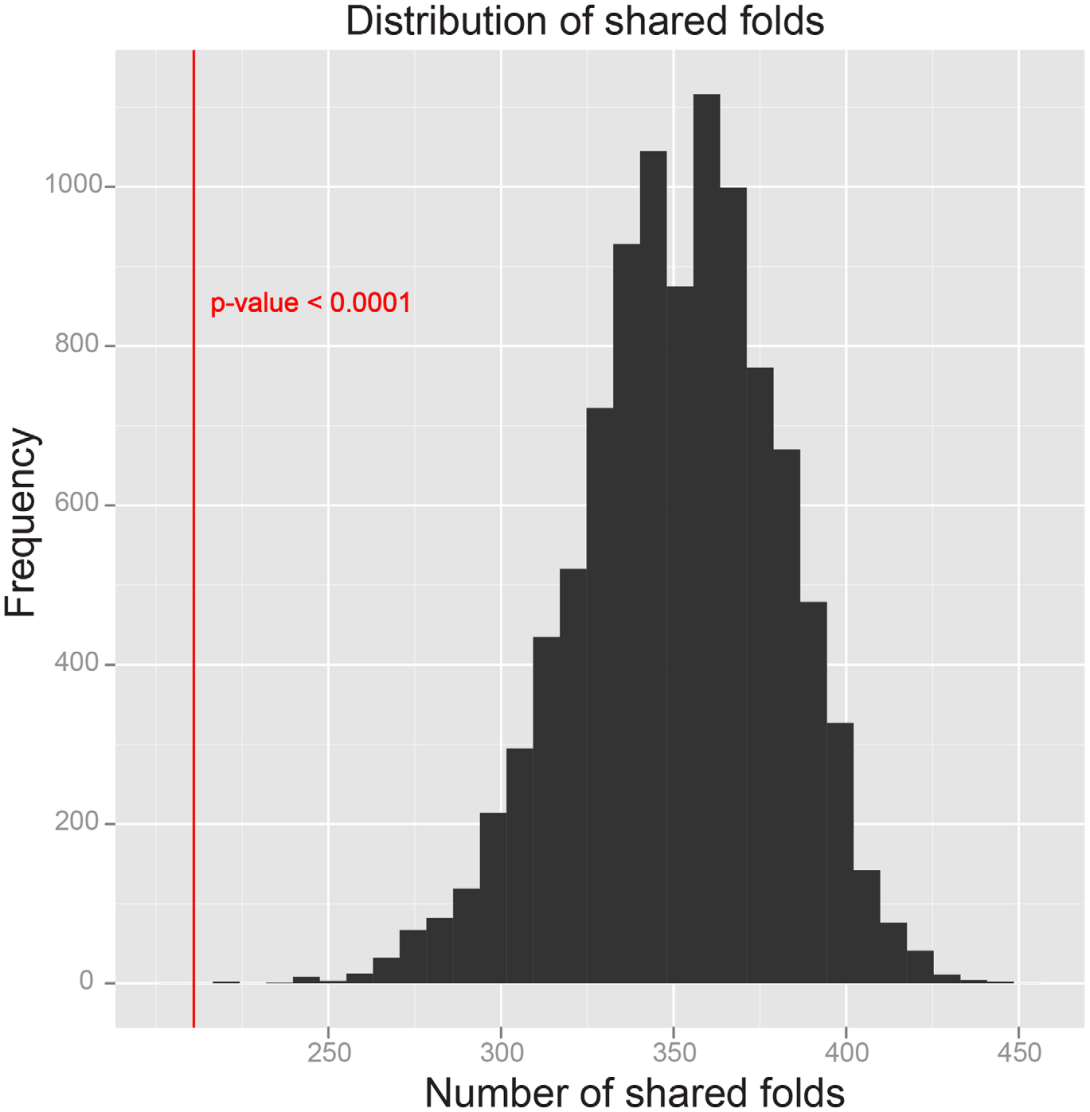
Statistical significance of test statistic. No single case in the 10,000 permutations has resulted in 210 or fewer shared folds between the set of 56 protein domains and their complement set, which makes the p-value of our test statistic less than 0.0001, as an upper bound for the statistical significance.

The distribution of shared folds, estimated from the 10,000 permutation tests, is plotted in Figure 7. The number of capsid-like folds shared by non-capsid proteins hence lies on the extreme left tail of the distribution, demonstrating that viral capsid folds are far less populated in structural fold space compared to generic proteins (Figure 7). The one-tailed p-value of our test statistic is less than 0.0001, and we thus conclude that there is significant statistical evidence against the null hypothesis that viral capsid folds span the protein fold space. We also show in Figure S1 (supporting information) that the p-value of our test statistic, based on the datasets containing domains of comparable sizes only, is 0.0002, therefore excluding size as a compounding factor contributing to the difference in fold. In conclusion, viral capsid folds are unique to viruses.

### Other functional classes

The seven other functional classes of proteins we examined range in size from 18 to 297 in the total set of 8921 proteins. When compared with their complementary set, the number of shared folds with non-self proteins is found to be statistically insignificant, with a one-tailed p-value greater than 0.05 in all cases (Table 2). This is not surprising, given that cellular proteins have evolved over a relatively shorter period of time, and therefore their folds are more similar to one another as compared to viruses, similar being defined by having a TM-score of greater than 0.4. We thus showed that it is not always true that unique function implies unique structural folds. Without making this assumption, we further proved that viral capsid proteins are segregated in structural fold space, which is remarkable.

**Table 2:** Seven functional classes of proteins we studied are found to be not significantly distinguished in their folded topology.

Functional class	Size of class	Subgroups, if any, included	One-tail p-value
Kinase	213	*-*	0.1449
Globin	32	*Myoglobin and hemoglobin*	0.4154
Dehydrogenase	297	*-*	0.3461
Polymerase	67	*DNA/RNA polymerase*	0.0572
Chaperone	33	*-*	0.2925
Antigen	49	*-*	0.4411
Muscle	18	*Actin, myosin, titin, nebulin*	0.1972

The shared folds between each functional class of proteins and their complement are not significantly small compared to what happens with random chances, with a one-tailed p-value greater than 0.05 in every case, suggesting that these cellular proteins are highly connected in structural fold space.

## Discussion

### Possible differences in domain definition

In this work, our major interest is to compare the independently folded domains of capsid proteins with generic protein domains, so as to reveal their relationship with the higher order of structural organization. Domains defined in this work therefore refer to integral structural units that are connected by single peptide to neighboring domains, although in a few cases these criteria are not fully met. We followed strictly the definition of domains in SCOP to make fair comparison with generic proteins collected from the same database. Our work does not focus on a finer granularity of structure such as subdomains, or motifs, which might have been called ‘domains’ in certain literature for the interpretation of their evolutionary origin. While our choice of domain definition addresses our question of interest adequately, we also note that the question of whether viral folds and generic proteins are evolutionarily segregated can be answered by comparing subdomains or structural motifs, which is outside the scope of discussion here.

### What are the capsid-like proteins and why they do not form shells

Prior to our work, several studies have reported that certain classes of cellular proteins also share similar topologies or structural cores with certain capsid proteins. These include the tumor necrosis factor superfamily [8], the serine proteases [13], the superantigen class [26], the concavalin A class [11], and the CUB-like domains [27]. All of the above classes of proteins were among the generic proteins that we found to share similar folds as capsid proteins, as expected. In addition, analysis of our set of 600 non-viral relatives of capsid proteins revealed that many virus proteases, certain hydrolases, transcription regulators and histone chaperones also shared close topological characteristics with viral capsid proteins (Table 1).

We first examined the structural relatives that are highly similar to capsid proteins (within a distance 0.4 or less). Many of these structural relatives possess the typical jelly-roll topology, with some variations in each case. The tumor necrosis factor superfamily is characterized by 10 strands in two sheets, with the core eight strands having identical connectivity as that of a standard capsid jelly-roll. Truncation in one strand and addition of two extra strands make them slightly different in shape compared to capsid proteins. The CUB-like domains in spermadhesins display a particular variation of the jelly-roll topology in terms of connectivity, including reversed β-strands, two disulphide bridges and two additional β-strands. They thus share a minimal structural core with capsid proteins (specifically the bean pod mottle virus capsid protein), but have shorter β-strands and overall smaller shape as a distinction. Superantigen Ypm is yet another class that overlaps significantly in structure with capsid proteins, especially satellite tobacco necrosis virus capsid proteins. Other than an additional disulphide bond connecting the C terminus with one β-strand that differentiates itself, superantigen Ypm also has a much more compact structure compared to capsid proteins, owing to its shorter loops connecting the β-strands. The supernatant protein factor protein consists of two domains, and the C-terminal domain also follows the jelly-roll topology that resembles satellite tobacco necrosis virus most, with minute differences in the concavity of the two β-sheets. The histone chaperone proteins are characterized by the same topology as capsid proteins, with some of them having one or two additional strands. Remarkably, all of these proteins discussed occur naturally (as opposed to crystal packing) as heterodimers (the monomers having identical topology), trimers, pentamers or hexamers, although their mode of interaction differ from that of capsid proteins in many cases. This suggests that the β-sandwich formed by proteins with varying connectivity generally facilitates aggregation, presumably because of the greasy, flat surfaces presented by their wedge-like shapes to promote monomer association.

In addition to these structural analogues found naturally in oligomeric states, we also identified quite a few proteins in the immunoglobulin fold and the methyltransferases fold that are highly similar to capsid proteins; however, they typically occur as part of some multi-domain proteins, such as the N-terminal binding fragment of the human polymeric immunoglobulin receptor. It thus might not be feasible to simultaneously arrange all domains on a shell in such cases, which may explain why we are not observing multimeric complexes for these proteins. We omit here discussion on the remaining types of protein domains, mainly for the reason of their limited structural similarity to capsid proteins (distance-wise more than 0.4 apart). These proteins typically either appear smaller in size or are tightly coupled with other domains, and consequently significantly different in shape, and have not been observed to form symmetric complexes in general.

Given the above interesting observations, we need to highlight that the structural relatives of capsid proteins only marginally resemble capsid proteins to the extent of their common structural core, as evident from the large structural distances (majority are greater than 0.4) between the two classes. Decorations on top of this level of similarity directly differentiate the exposed edges of the proteins, such that geometrical complementarities along multiple symmetry axes are easily satisfied by repeating units of the same monomers in the case of capsid proteins but not in the other. In other words, the positions in which monomers interact with one another are also fine-tuned by geometric and physicochemical factors of protein-protein interfaces. We thus do not observe any protein cages assembled from these cellular proteins despite their sharing similar structural topologies with capsid proteins. Lastly, we speculate that the structural but not functional close relationship between these few classes of proteins and capsid proteins resulted from ancient genetic interactions between viruses and their hosts, although further investigation is needed to support this view.

### Scarcity and possible bias in the data

An important aspect that cannot be overlooked is that we have drawn our conclusion in this work based solely on existing structural data of capsid proteins taken from icosahedral viruses. We cannot exclude possibilities of identifying novel viral capsid folds that span a larger subspace of protein folds in future, as predicted in several recent publications [16], [17], [28] given the diversity of the virosphere. This is especially so when we take into account the current challenges in determining the structure of viral proteins embedded in lipid membranes for enveloped viruses. In addition, experimental limitations in determining the structure of large assemblies place a heavy bias in highly symmetrical viral particles, and thus statistics for irregularly shaped viruses such as HIV are missing in our analysis. Given all structural data available up to this date, we have derived our conclusion with rigor and confidence, but we remain open to potential changes should abundant novel discoveries be made.

### Implications on protein-protein interaction and other applications

Our study provided support for the hypothesis that viral capsid proteins, which are functionally unique in viruses in constructing protein shells, are also structurally unique in terms of their folding topology. This implies that protein-protein interactions, in the case of viral capsids at least, confer evolutionary constraints on capsid proteins, specifically on their folds. Bhadur and Janin [29] found that residues making up capsid cores are more conserved than interface residues and surface residues, which highlights a greater selective pressure on capsid structural core. Interpreted together, the characteristic folds (and therefore fundamental shapes) of capsid proteins are most likely a consequence of geometric requirements of the building block so as to form the cage-like macromolecular assembly, which corroborates the theory proposed by Mannige and Brooks [30]. From a more general point of view, core residues of cellular proteins have also been known to be evolving at a slower rate compared to interface and surface residues [31], with a 25%–35% higher conservation score compared to surface residues. Most studies that investigated the degree to which proteins are subject to constraints due to their interactions with other proteins mainly focused on interface residues [31]–[33], and it remains to be established whether the greater conservation of structural cores of generic proteins is similarly affected by the interaction with their partners during evolution. Our work sheds light on this missing link by studying the particular case of viral capsid proteins, and it will be interesting to verify whether this evolutionary constraint is true in general.

Additionally, virus-like particles (VLPs), which are self-assembling capsid shells without the infectious viral genetic materials encapsulated, are already a popular choice among a variety of nanoparticle platforms for wide applications both in the biomedical arena and in material science [34]–[40]. For a comprehensive review, readers may refer to this paper [41]. Compared to other nanoparticle materials, VLPs offer several advantages, including the full range of protein templates they provide that adapt to diverse environmental conditions including extreme thermal environments [42], their proteinaceous nature which makes them biodegradable [43], and their plasticity to a wide range of synthetic manipulations [44]–[46]. For biomedical applications, VLP design has been formulated for targeted delivery of drug molecules [47], tissue-specific imaging reagents [45], as well as novel vaccine development [48]. VLPs have also been extensively explored as nanocontainers [49] and nanotubes [50] in materials science. In order to fulfill their desired purposes, VLPs are introduced new functional modules, to facilitate specific interactions with the intended biological sites or nonbiological surfaces, to alter the overall architecture and stability [51], and to package various cargos as well as directing the cage assembly [52]. Our work laid out the fundamental principle in such tailored design of VLP platforms; in order to preserve the assembled architecture of viral capsid shells, it is important for the newly formulated protein subunits to adhere to the library of viral capsid folds. In other words, significant adaptations that result in unfolding or misfolding of capsid proteins are undesirable. Where human creativity has no bound in exploring all synthetic possibilities, feasibility has its bound; decorations on VLPs should minimally disrupt the folded topology and geometry of the building block to make it work.

### Are viral capsid interfaces also unique to viruses?

Having established that viral capsid proteins possess distinct folds, we would like to take one step further by examining whether the protein-protein interfaces in viral capsid assemblies are also unique to viruses. Because differences in monomer structure do not imply differences in protein-protein interfaces [22], our conclusion of the uniqueness of capsid fold cannot be directly extended to capsid interfaces. The results of this second comparison will again have interesting implications. Should capsid interfaces resemble those of generic ones, the mode of capsid-capsid interation is then governed purely by physicochemical laws, and evolution merely plays a part in dictating the building block structure for their proper tiling. If, on the other hand, we learn that viral capsid interfaces are quantitatively different from interfaces formed by their cellular counterparts, we can then tap on this difference and design pathogen-specific antiviral drugs targeted at disintegrating the protection shells, without disrupting normal cellular activities. Work in this direction is in progress.

In summary, our comprehensive analysis of the viral capsid proteins and their cellular counterparts revealed the segregation of capsid proteins in structural fold space. This provides important clues to requirements of the building blocks for the distinctive viral shell architecture; the unique folds of viral capsid proteins present favorable geometry to allow effective packing and assembly into the right complex architecture. With this in mind, the design of gene therapy delivery agents as well as nanoparticles, both targeted at making packing tools, can be tailored to satisfy geometric constraints by following closely the viral capsid templates nature has created for us.

## Supporting Information

Figure S1 **Statistical significance of test statistic for domains with fewer than 600 residues.** In order to compare capsid and non-capsid sets that are of comparable sizes, the same analysis was applied to the two sets less those domains greater than 600 residues in length. The p-value obtained for our test statistic of 210 capsid-like folds is 0.0002, which is evidence that with the bias in protein sizes removed, the two sets still have different folds with statistical significance. Hence this suggests that size is not a major factor contributing to the uniqueness in folded topology of capsid proteins.
(TIF)

Table S1 **Capsid proteins added from SCOP that were not deposited in VIPERdb.** These 24 proteins were correspondingly removed from the non-capsid set and added to the capsid set, before structural clustering was performed.
(DOCX)
