# Supplementary figures and images for: Viral Capsid Proteins Are Segregated in Structural Fold Space
Source: PLoS Comput Biol. 2013 Feb 7;9(2):e1002905. doi: 10.1371/journal.pcbi.1002905 (PMC3567143; doi:10.1371/journal.pcbi.1002905)

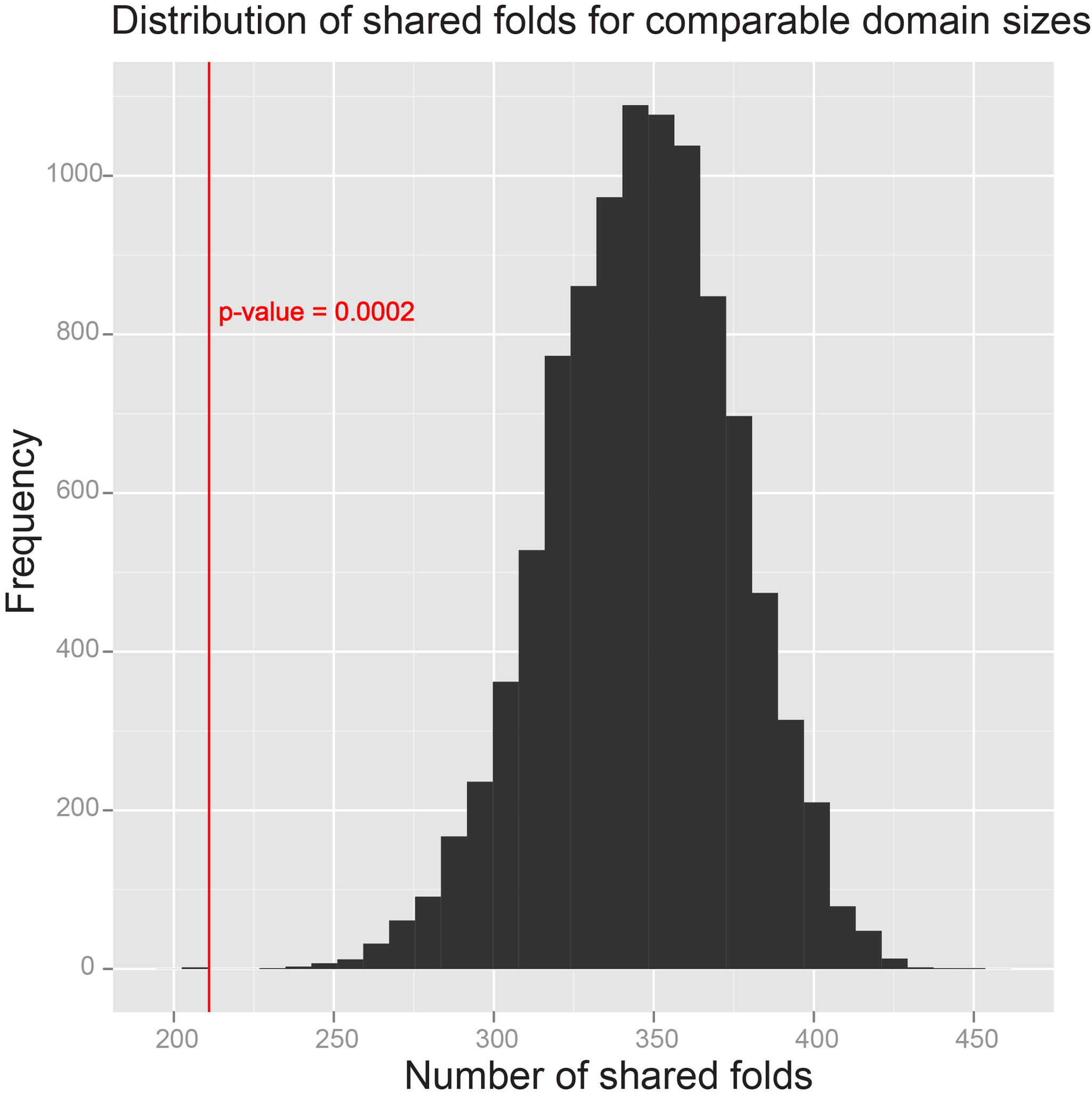

Supplement: Figure S1 — Statistical significance of test statistic for domains with fewer than 600 residues. In order to compare capsid and non-capsid sets that are of comparable sizes, the same analysis was applied to the two sets less those domains greater than 600 residues in length. The p-value obtained for our test statistic of 210 capsid-like folds is 0.0002, which is evidence that with the bias in protein sizes removed, the two sets still have different folds with statistical significance. Hence this suggests that size is not a major factor contributing to the uniqueness in folded topology of capsid proteins. (TIF) [file pcbi.1002905.s001.tif]
