# Supplementary material for: Viral Capsid Proteins Are Segregated in Structural Fold Space
Source: PLoS Comput Biol. 2013 Feb 7;9(2):e1002905. doi: 10.1371/journal.pcbi.1002905 (PMC3567143; doi:10.1371/journal.pcbi.1002905)
Supplement: Table S1 — Capsid proteins added from SCOP that were not deposited in VIPERdb. These 24 proteins were correspondingly removed from the non-capsid set and added to the capsid set, before structural clustering was performed. (DOCX) [file pcbi.1002905.s002.docx]

Table S1. Capsid proteins added from SCOP that were not deposited in VIPERdb.

| _SCOP id_ | _Description of protein_ |
| --- | --- |
| _d1wyka__ | _Sindbis virus capsid protein_ |
| _d2df7a1_ | _structural polyprotein VP2_ |
| _d1vpsa__ | _Polyomavirus vp1 pentamer_ |
| _d1hx6a2_ | _major capsid protein_ |
| _d1p2za1_ | _Hexon protein_ |
| _d1p2za2_ | _Hexon protein_ |
| _d1ahsa__ | _African horse sickness virus (serotype 4) vp7_ |
| _d1bvp11_ | _bluetongue virus coat protein vp7_ |
| _d1bvp12_ | _bluetongue virus coat protein vp7_ |
| _d1qhda1_ | _viral capsid vp6_ |
| _d1qhda2_ | _viral capsid vp6_ |
| _d1jmu.1_ | _protein mu-1_ |
| _d1u7ka__ | _Gag polyprotein_ |
| _d2eiaa2_ | _Eiav capsid protein p26_ |
| _d1em9a__ | _Gag polyprotein capsid protein p27_ |
| _d1tx9a1_ | _Scaffolding protein D_ |
| _d1kqra__ | _VP4_ |
| _d1svba2_ | _Tick-borne encephalitis virus glycoprotein_ |
| _d2alaa2_ | _Structural polyprotein (P130)_ |
| _d1slqa__ | _VP4_ |
| _d1m3ya1_ | _The Major capsid protein of PBCV-1, Vp54_ |
| _d1m3ya2_ | _The Major capsid protein of PBCV-1, Vp54_ |
| _d2pxrc1_ | _Gag-Pol polyprotein (Pr160Gag-Pol)_ |
| _d2v33a1_ | _E1 envelope glycoprotein_ |
